# Occupational Blast Wave Exposure During Multiday 0.50 Caliber Rifle Course

**DOI:** 10.3389/fneur.2019.00797

**Published:** 2019-07-25

**Authors:** Maciej Skotak, Christina LaValle, Anthony Misistia, Michael J. Egnoto, Namas Chandra, Gary Kamimori

**Affiliations:** ^1^Department of Biomedical Engineering, New Jersey Institute of Technology, University Heights, Newark, NJ, United States; ^2^Blast-Induced Neurotrauma Department, Walter Reed Army Institute of Research, Silver Spring, MD, United States; ^3^Geneva Foundation, Tacoma, WA, United States; ^4^Cherokee Nation Businesses, Catoosa, OK, United States

**Keywords:** low-level blast, occupational exposure, peak overpressure, repeated blast exposure, neurocognitive performance

## Abstract

Research on blast overpressure (BOP) experienced by military personnel in operations like breaching, identifies transient, measurable effects on operator readiness. Specifically, blast seems to be associated with suppressed response speed and cognitive function. This work evaluates 50 caliber weapon systems to ascertain BOP effects from the weapon usage. Marksmen were a collection of professionals who use 0.50 caliber weapon systems as part of their daily activities, and the environment measured was during a training course. The 20 human subjects were equipped with B3 blast gauges and occupational BOP exposure monitored over the course of 3 day training period with measurements taken from 500+ shots. We noted a considerable variation in total cumulative peak pressure (50–350 psi) and impulse (25–180 psi·ms) values. The frequency analysis (number of shots fired by the trainee) revealed that the number of exposures per day varied between 4 and 27 per day (peak at 7: 14.3% of the data), and 2 to 17 per hour (peak at 8: 18% of the data). The cumulative number of exposures was 24–50 per trainee. The neurocognitive performance was evaluated using Defense Automated Neurobehavioral Assessment (DANA) Rapid: Simple Reaction Time (SRT), Procedural Reaction Time (PRT) and Go/No-Go (GNG). The results recorded before the training were a baseline for each training day and compared with the results recorded after and at the end of the day. Only PRT and GNG tests revealed a cumulative increase in proportion of subjects with slowed reaction times over the progression of course with concomitant dispersion increase at the end of the day. Noticeably, on average 2/3rd of the trainees performed faster, while 1/3rd of trainees performed these tasks slower, but there was no correlation with the cumulative pressure dosage. The fatigue appears as an aggravating factor affecting the neurocognitive performance, and a more sophisticated evaluation regimen is necessary to discern potential neurological effects. Additional investigation is needed to understand the increasing dispersion of results between subjects and future works should be mindful of such continued trends. Future work should seek to determine the recovery period and longitudinal effects of heavy usage of these weapon systems.

## Introduction

Repeated exposure to low-level blast (LLB) was recently a subject of an expert panel proceedings which was identified as a next generation challenge in military medical research (1). Low-level blasts occur in standard training paradigms during the use of explosives and weapons by military or law enforcement personnel. Unlike civilian mild Traumatic Brain Injury (mTBI), which is diagnosed as a clinical injury, the set of similar diagnostic tools is currently not available to determine the extent of neurological effects associated with LLB (2). More importantly, there exists a knowledge gap correlating immediate and long-lasting effects of repeated exposure to overpressure waves on the human brain, which would serve as a basis for the development of the diagnostic tools (3).

The type of exposure prevalent in training in many military and law enforcement occupations (e.g., artillery, mortars, heavy weapons, explosive breaching), is characterized by magnitude of overpressure and impulse values which are typically lower when compared to explosive exposures (4–6). Other characteristics of repeated occupational LLB which distinguish it from higher-level blast experienced in a combat setting are: (1) high rates of consecutive repetitions in non-combat training protocols, (2) highly variable duration of periods between consecutive exposures, and (3) the duration and intensity of training usually only lasting for a few consecutive days. The existing studies suggest the existence of adverse neurological effects which might lead to the removal from active service (3). A general consensus is that the evidence correlating LLB with neurological effects is limited and anecdotal (self-reported), which suggests the association between LLB and neurologic dysfunction is possible (7), but there is no reliable evidence to support or overturn this hypothesis (3). The early confirmation connecting the LLB with neurological abnormalities are the studies on breachers, which were reported more than a decade ago by operating independently military medical providers from multiple countries (2). The cluster of subjective symptoms described as “breacher's brain” including a headache, fatigue, a slowed thought process and an increase in memory deficits bear a striking resemblance to symptoms of concussion (2, 8–10). These reports led to several blast exposure studies which focused on outcome measures (e.g., biomarkers, neuroimaging, cognitive performance, and symptom reporting). However, these works lacked accurate individual blast exposure measures (2, 8, 11, 12). It is important to note that although significant physiological changes were identified in some of these studies, the effects were transient and none among affected individuals were diagnosed with an injury (i.e., concussion). For example, an anonymous survey of self-reported symptomology among a cohort exposed to explosive blasts in training was used to explore these effects (9). A headache, difficulty sleeping, irritability, cognitive impairment, and a variety of other symptoms consistent with post concussive syndrome (PCS) were reported more often and at greater severity by those exposed to blast (sample size *N* = 135) than by a comparison cohort not exposed to blast (*N* = 49). Specifically, among the set of 35 symptoms examined, the average number reported by the cohorts exposed vs. not exposed to blast was 8.7 and 5.2, respectively [independent samples *t*-test: *T*_(182)_ = 2.18 with *p* = 0.030]. In another study, 33 US Marines were instrumented with pressure gauges (helmet and shoulders) during a 2 week training protocol including explosive breaching and a variety of breaching charges (13). Researchers administered neuropsychological assessments across the training period, including computer-based neurocognitive assessments and fMRI during memory and word retrieval tasks. In comparisons between soldiers before and after blast exposure, negative effects were observed following a blast in reported-headache and in task performance involving working memory, but these effects were observed only in the instructor group which consequently had the highest blast overpressure (BOP) cumulative dosage.

Baker et al. (11) conducted a separate study of effects following LLB exposure in a Canadian law enforcement unit. As in the study of US Marines, the Canadian study used a prospective observational design with neurocognitive and vestibular measures in a training setting for explosive breaching. That study also included less experienced participants (*N* = 10) along with a small group of more experienced participants (*N* = 4). Unlike the US Marines study, the Canadian study did not reveal post exposure differences for either group of participants. A key difference between these two studies was that the wearable overpressure sensors used by the US Marines showed blast exposures with peak overpressure values as high as 13 psi (90 kPa), whereas exposures in the Canadian sample were estimated as not exceeding 3 psi (21 kPa). Small sample size might also contribute to the lack of sensitivity in this evaluation.

The current lack of clearly documented neurologic effects and effective measurement methods for those effects hinders the evaluation and mitigation of neurotrauma risk from LLB (3). The standard training protocols involving blast restrict the exposure to a limit of 4 psi (28 kPa) peak overpressure, an exposure limit designed to decrease the potential for damage to the unprotected human eardrum rather than damage to the brain. There are no guidelines regarding the total allowable dose, expressed as the number of exposures per training session, that a single trainee can experience without detrimental effects on health and neurological performance. Moreover, the safe-distance estimate, which is based on an open-field explosion, was shown to be underestimated in the confined space typical for high-explosive indoors breaching (3, 5). The existing base of knowledge is limited, and results are frequently hard to interpret due to a variety of exacerbating factors, including relative novelty of the occupational blast exposure problem as a health hazard and the inconsistency of findings (3).

This paper describes the complexity of the training environment of the 3 day long 0.50 cal sniper rifle marksman course. The trainees were equipped with a set of four (left and right wrist, shoulder, and head) Blast Gauge^®^ generation 6 pressure sensors each to monitor the environmental exposure. We report peak overpressure, impulse and several variables detailing the frequency of exposure to the blast. We believe the characterization of the exposure conditions is an important factor to delineate the neurological effects and that similar level of detail was not demonstrated before in the available literature.

## Materials and Methods

### Test Subjects

A total of 20 marksmen participated in the study: 13 subjects used 0.50 caliber sniper rifle equipped with 29” long barrel, while 7 subjects used 20” long barrel for the duration of the training. Subjects were a collection of professionals who use 50-caliber weapon systems as part of their daily activities, and the environment measured was during a 50-caliber training course. The course ran for 3 days at a private facility in the Southern United States and was restricted to military and law enforcement professionals. Participants were all male and ranged in age between late 20s to early 50s. Due to the sensitivity of some of the attendee's work, more specific demographics could not be collected.

### Blast Exposure Monitoring and Neurocognitive Testing

**Figure 1:**
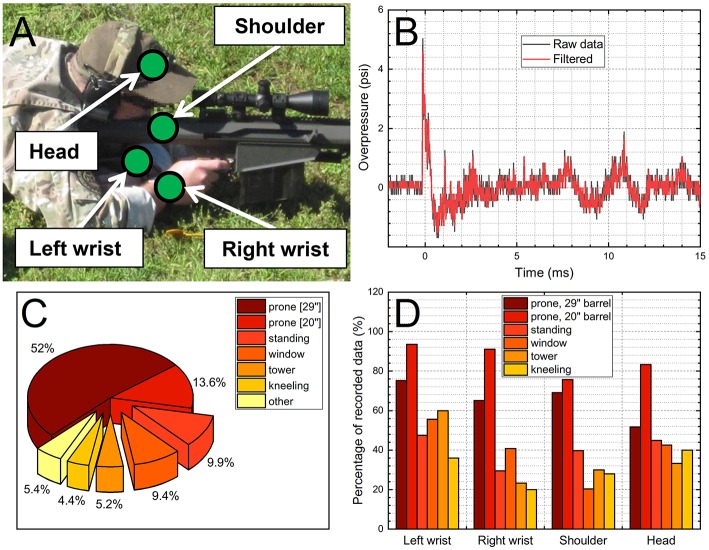
The 0.50 caliber rifle training: **(A)** schematic of the sensor locations worn by the training participants, **(B)** example of the overpressure data collected by a B3 sensor (raw and filtered data are presented), **(C)** the pie chart illustrating fractional amount of the overpressure data collected during the 3 day course expressed as various shooting positions, **(D)** the percentage of the collected data as a function of the sensor location and shooting position.

The blast gauge sensoring scheme used in this study is depicted in Figure 1A: the trainees had a set of four sensors attached during 3 days of exercise—two on the left and right wrists, one on the head and one on the shoulder. The same set of four sensors was worn by an individual trainee for the duration of the course, and the same sensor was used in the designated mounting location. Sensors were configured in such a way to trigger data recording by the pressures as low as 2.5 psi. The Blast Gauge^®^ (BlackBox Biometric; Rochester, NY) generation 6 pressure sensors record the data for 20 ms with 100 kHz sampling frequency (representative raw and filtered pressure profiles are presented in Figure 1B), which is sufficient to capture pressure wave characteristics, peak overpressure, duration and impulse (14).

The two weapon systems with different barrel lengths (20-inches and 29-inches, respectively) were used with measurements taken from over 500 shots. The number of exposures per trainee were calculated using time stamps recorded by the B3 blast gauges and verified independently with a shot log, where number of rounds fired were counted and recorded by research team members. Generally, ammunition used was either M33 ball or was Hornady 750 gr A-Max (Jacketed boat-tail ballistic-tip HP). The cumulative BOP and impulse values were calculated using left wrist sensor data, considering this sensor had the highest success rate of the pressure recording (Figure 1D). The average BOP and impulse values were multiplied by the number of exposures per trainee and gave the cumulative BOP and impulse values. Before, immediately after (except for day 1) and at the end of the courses of fire during each of the 3 training days, participants were administered the DANA Rapid module, comprised of 3 subtests, simple reaction time (SRT), procedural reaction time (PRT), and Go/No-Go (GNG; response inhibition) (15). SRT: the subject taps on the location of the yellow asterisk symbol as quickly as possible each time it appears. This task measures pure reaction time. PRT: the screen displays one of four numbers for 3 s. The subject presses on a left button (2 or 3) or right button (4 or 5) depending on the number pressed. A choice reaction time measure of accuracy, reaction time, and impulsivity. This choice reaction time task targets simple executive functioning with easy decision-making capabilities. GNG is a forced choice reaction-time task relevant to warfighters. A house is presented on the screen with several windows. Either a “friend” (green) or “foe” (white) appears in a window. The respondent must push a “fire” button only when a “foe” appears. A choice reaction time measure of sustained attention and impulsivity. The test assesses speed and accuracy of targets, omissions, and commissions. DANA Rapid was used to evaluate the performance of the trainees and it was administered on: (1) Day 1, at 8:00 (pre) and 15:00 (post), (2) Day 2, at 6:30 (pre), 9:00 (immediate), and 15:00 (post), and Day 3, 6:30 (pre), 11:0 (immediate), and 13:30 (post). We evaluated the data by using the scores recorded at the beginning of on each training day (pre) as a baseline. These test results were also used to construct the differential values in respective tests, i.e., the “pre” score values were subtracted from the “immediate” or “post” values giving the corresponding ΔSRT, ΔPRT, and ΔGNG values (for a specific trainee).

The results of DANA Rapid were separated into two groups “slower” and “faster” based on the intraday and end-of-day performance corrected for the baseline morning readout. The result was classified as “slower” if the value was more than zero indicating that the reaction time decreased after the course, or it was classified as “faster” when the value was <0, which indicates improvement in the reaction time. The average values for both groups were calculated, plotted as a function of time and subjected to linear regression analysis.

The data presented in the quadrant plots were calculated as follows: the baseline for a specific day (1, 2, or 3) were tests performed before the training session, and two differential values were considered: using test results for the immediate test session, and test results obtained after the day of training. Similarly, the cumulative BOP values are the “daily dose” recorded for a specific day. The “immediate” tests were not performed on day 1 of the training, and effectively these quadrants have only 40 data points (2 days × 20 trainees = 40) vs. 60 data points for the “post” test result plots. The quadrants were constructed as follows: the zero line indicates no change in the performance of the trainee in the PRT or GNG tests (the SRT test was eliminated from this analysis because it didn't show any changes in the analysis of the cumulative SRT values). The 70 psi is 50% of the highest recorded cumulative daily dose (140 psi BOP).

### Statistical Analysis

The data were tested for homoscedasticity, normality (using Ryan-Joiner test) and outliers using Minitab 18.0. The unpaired two-tailed *z*-test with Bonferroni correction for multiple comparison was performed for intra- and inter-group comparison of the blast gauge data, and *p* = 0.008 was assumed as a threshold of statistical significance. The repeated measure *t*-test with multiple comparison Bonferroni correction was performed on average SRT, PRT, and GNG intraday values, and *p* = 0.025 was assumed as a threshold of significance. Power analysis was performed with GPower 3.1.9.2 software with α values of 0.008 and 0.025, for blast gauge and DANA Rapid data, respectively. Data are presented as average ± standard deviation. The linear regression of the ΔSRT, ΔPRT, and ΔGNG data as a function of time was performed in Minitab 18.0. The 08:00 am on the day 1 of the training is used as a reference.

## Results and discussion

### Evaluation of Overpressure Dosage During Training

Most of the occupational exposure evaluation was performed for the combination of prone position and 29” barrel (298 measurements, which is 52% of the total data sets recorded during training, Figure 1C). Prior 0.50 caliber investigations indicate that the prone shooting position is both the most commonly used position and the most likely to create the highest measurable levels of the BOP (16). The percentages of the other positions were: 13.6% (prone, using 20-inch barrel), 9.9% (standing), 9.4% (window), 5.2% (tower), 4.4% (kneeling), and 5.4% recorded in other positions. These percentages use the total number of tests as a denominator, and it is obvious that the percentage of retrieved pressure data vary significantly depending on the sensor location and test configuration (Figure 1D). In general, the highest percentages were recorded when the shorter, 20-inch barrel was used (between 78 and 97%) but were as low as only 20% for the window and kneeling positions for right wrist and shoulder sensors, respectively.

**Figure 2:**
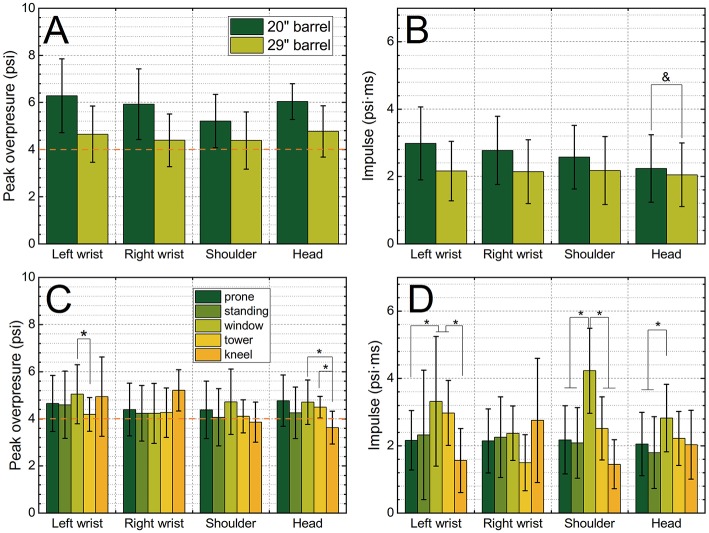
The average peak overpressure **(A,C)** and impulse **(B,D)** as a function of sensor location (left wrist, right wrist, shoulder, and head). The comparison of the peak overpressure and impulse for the 20-inch and 29-inch barrel **(A,B)** indicates deployment of a shorter barrel results in increased pressure exposure (*p* < 0.005, ampersand indicates the only pair of groups where there were no statistically significant differences). The comparison of average peak overpressure **(C)** and impulse **(D)** values recorded when 29-inch barrel was used during the training, in five shooting configurations: prone, standing, window, tower and kneeling. Groups exceeding statistical significance threshold *p* < 0.005 are marked with asterisk.

The effect of the barrel length on the occupational pressures recorded by four sensors for prone position is depicted in Figures 2A,B. The recorded peak overpressures are higher for the shorter barrel, compared to 29-inch barrel: 5.85 vs. 4.55 psi (average across all four sensors), respectively. All four sensors reported peak overpressure values which are higher for shorter barrel length, and these differences are statistically significant (*p* < 0.005), with the effect size ranging between 0.65 and 1.28, and power of 0.99. Similar trends are observed in the impulse values, except for the head sensor where values for both barrels are not statistically significant (Figure 2B, marked with ampersand). We performed the comparison of the effects associated with five different training positions (prone, standing, window, tower, and kneel) where only 29-inch barrel was used, and the results are presented in Figures 2C,D. The variability between peak overpressure values is rather small with just a few statistically significant results (Figure 2C, marked with asterisk). Larger variability is seen in the impulse values, which is partially due to the integration algorithm embedded in the sensor, which takes the entire 25 ms of the recorded signal. It results in the larger variability of the calculated impulse values depending on the baseline signal fluctuations.

### Reliability of Pressure Measurements

The complete data set from a single measurement should consist four peak overpressure and four impulse values. However, we noticed that this is hardly the case for most of the measurements. Consequently, many measurements are necessary to obtain reliable data: among 298 measurements performed for 29-inch barrel in prone position the full set of 4 BOP values was recorded in only 28.2% cases, and the remaining success rates were 33.9% (3 BOP values), 18.1% (2 BOP values), 10.1% (1 BOP value), and 9.4% (0 BOP values). For the 20-inch barrel the success rate distribution is as follows: 59.5% (4 BOP values), 29.1% (3 BOP values), 6.3% (2 BOP values), 1.3% (1 BOP value), and 3.8% (0 BOP values).

**Figure 3:**
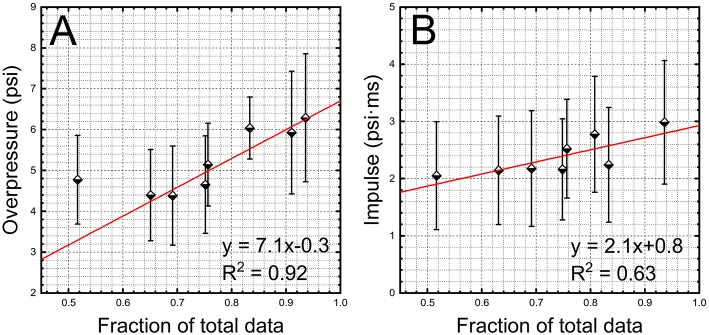
The results of the linear regression analysis on peak overpressure **(A)** and impulse **(B)** values. The data collected for 20-inch and 29-inch barrels separated by a sensor location were pooled together. The average values of BOP and impulse as a function of the fraction of the recorded data were used to perform linear regression. The sensors used to monitor the occupational exposure are gradually losing their sensitivity (ability to trigger data recording) at lower BOPs.

It would appear the higher success rate depends on the pressure intensity. To test this hypothesis, we performed the regression analysis using pressure data recorded in prone position where 20- and 29-inch barrels were used. The data for each sensor were pooled together, averaged and plotted as a function of the fraction of the recorded data (Figure 3). Indeed, the linearity between the pressure intensity and fraction of the recorded data is clearly demonstrated in Figure 3A, with a high regression coefficient value, *R*^2^ = 0.92 (the outlier in this data set is a head sensor with mean of 4.77 psi and only 0.52 fraction of recorded data). The same analysis performed on impulse values, results in less satisfactory results (*R*^2^ = 0.62, Figure 3B).

### Cumulative Number of Exposures and Overpressure

**Figure 4:**
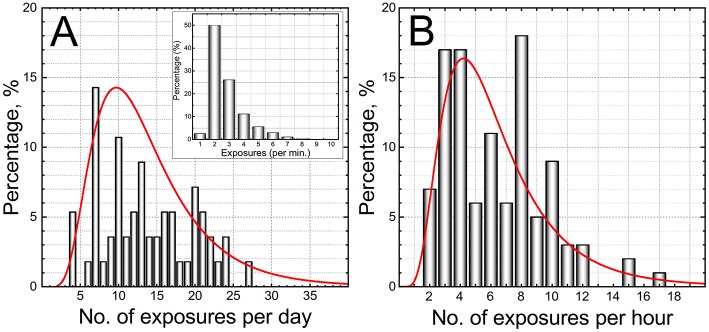
The analysis of the number of exposures illustrates the dynamics of the training environment experienced by trainees. The frequency of the number of exposures: **(A)** per day, and **(B)** per hour are presented. All data of the 3 day training course were pooled together irrespectively on the rifle configuration or shooting position. The inset in panel **A** is a histogram which represents the frequency of the “burst” exposure during the training in the shooting range.

The 0.50 caliber training course is an environment with highly heterogeneous occupational exposure conditions. The number of rounds per trainee is highly variable. The number of fired rounds will directly translate to the cumulative overpressure dose, and thus it is imperative to evaluate the number of exposures per time in training. Our analysis indicates the number of exposures per day, per trainee ranged between 4 and 27 (Figure 4A), while the number of exposures per hour per person is in the 2 to 17 range (Figure 4B). Moreover, we noted the number of consecutive rounds fired within a 1 min period was in the 1 to 8 range (inset, Figure 4A).

**Figure 5:**
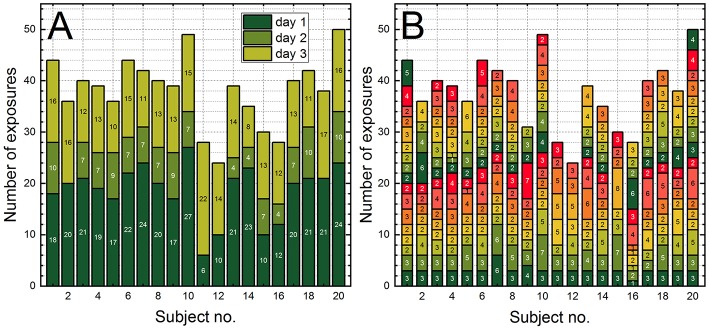
The stacked column bar plots illustrating the cumulative number of exposures per trainee expressed as: **(A)** the sum of exposures per day, and **(B)** the sum of consecutive exposures recorded by the pressure sensors within 1 min (the “burst” exposure). The sum of “burst” exposures in **(B)** corresponds to the daily (counting from the bottom to the top) and the total sum of exposures in **(A)**.

The data sets with significant loss of BOP and impulse values could be used for estimation of the cumulative number of exposures during the 3 day course. Our analysis relies on the two methods: the log of number of shots fired by trainee, and the time stamp corresponding to the event as an indicator of the exposure, irrespectively how many data points were retrieved from the four sensors. The results of the cumulative number of exposures per trainee are presented in Figure 5. In the Figure 5A, the total number of exposures is expressed as a sum of daily number of exposures, while in Figure 5B the same total number of exposures is expressed as a set of 1 min “burst” exposures. Overall, the cumulative number of exposures per trainee ranges between 24 and 50.

**Figure 6:**
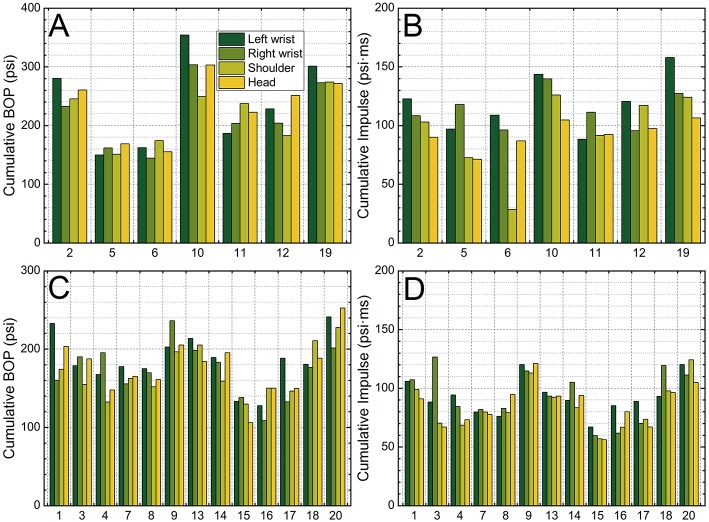
The cumulative peak overpressure **(A,C)** and impulse **(B,D)** values per trainee. There are two groups of trainees, who used a 20-inch barrel **(A,B)** or 29-inch long barrel **(C,D)** during the training. The cumulative exposure is defined as number of exposures per day multiplied by the average peak overpressure or impulse value.

In the next step we estimated the cumulative “dose” per trainee based on the average peak overpressure and impulse values collected using all shooting positions. For this purpose, the average daily BOP (or impulse) obtained from the left wrist sensor was multiplied by a daily cumulative number of exposures (Figure 5) and the sum over 3 days was calculated. The left wrist sensor reported the highest rates of recorded pressures (Figure 1D), and the application of these values is a method to diminish the uncertainty in the estimation of total dose. The results are presented in Figure 6. The data are separated based on the barrel length: in Figures 6A,B the cumulative BOP and impulse, respectively, is presented for 20-inch long barrel, while in Figures 6C,D, the analogous cumulative BOPs and impulse values are presented for the trainees using 29-inch long barrel. It is noteworthy that relatively large variability in cumulative values which exists between sensors worn by the same person, e.g., for the trainee no. 1 the cumulative BOP estimate varies between 160 and 233 psi (Figure 6C).

### Neurocognitive Performance Evaluation

**Figure 7:**
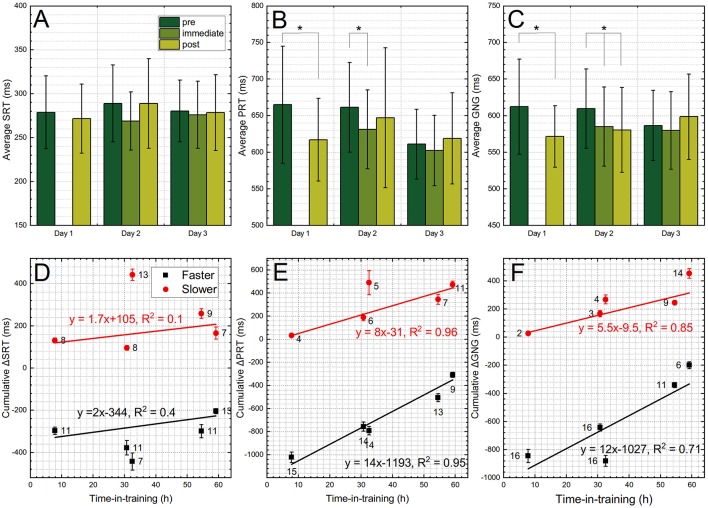
The results of the DANA Rapid tests. The average cumulative values of the: **(A)** SRT, **(B)** PRT, and **(C)** GNG modules. These tests were performed before the training (pre), immediately after the training (immediate) and after the training (post). The data for each test were normalized by subtracting the values recorded before the training and separated as a “faster” and “slower” when these values were higher or below the zero, respectively. Then the data in respective groups were pooled together and plotted as a function of the training day for: **(D)** ΔSRT, **(E)** ΔPRT, and **(F)** ΔGNG test modules. The gradual shift toward the “slower” group, both in terms of number of trainees (numbers above or below of the data points) and the cumulative values (bar length) is observed with the time of training for PRT and GNG tests. Groups exceeding threshold of statistical significance (*p* < 0.005) are marked with asterisk.

The average SRT results indicate (Figure 7A) there were no statistically significant differences between any day of training, but the same is not true for the PRT and GNG tests (Figures 7B,C, respectively), where statistically significant results were detected on the days 1 and 2, but not day 3. These results would indicate that on average the trainees performed the reaction time tasks faster after the training on days 1 and 2. However, to gain better insight we separated the results on the “faster” and the “slower” groups (Figures 7D–F). For that purpose, the differential values were used, i.e., if the trainee performed a task faster than the baseline (the “before” test session), the score would be negative, while the opposite is true for the slow responders, who get a positive value. When we separated these two groups and calculated the sum of the differential values per test, it become apparent that for the ΔPRT and ΔGNG tests, the cumulative values in the “faster” group gradually decrease and are strongly correlated with the time (*R*^2^: 0.95 and 0.96 for ΔPRT, and 0.71 and 0.85 for ΔGNG, Figures 7E,F). This trend is caused by gradually decreasing number of trainees in the “faster” group, with simultaneous increase of the “slower” population, as indicated by numbers annotated next to the symbols (Figures 7E,F). It might indicate the fatigue is a factor in these tests, which might mask the behavioral effects, or these tests are not sensitive enough to probe acute neurological effects associated with repeated occupational LLB. Interestingly, the fatigue was the most frequently self-reported symptom (18 out of 19) among breachers (2). Our previous studies on benefits of caffeine in the prolonged sleep deprivation during military exercises, indicate that the fatigue correlates with the time, observed as a decrease in Psychomotor Vigilance Test (PVT) performance in placebo group (17–19).

**Figure 8:**
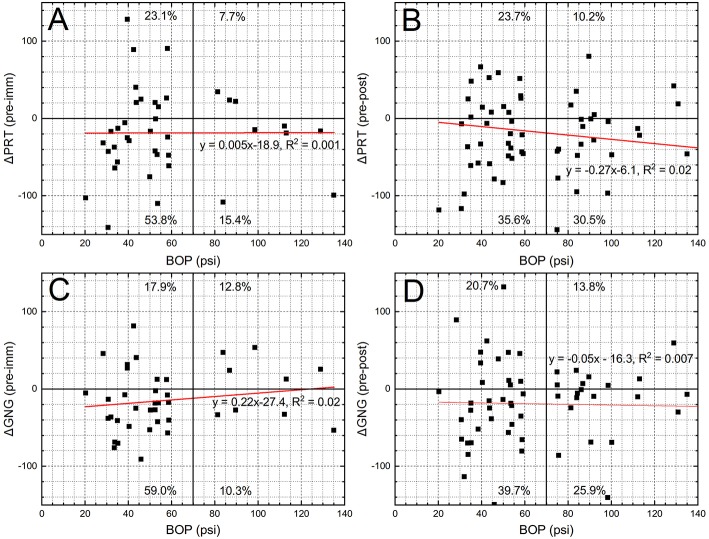
The ΔPRT **(A,B)** and ΔGNG **(C,D)** data were pooled from 3 days of the training and expressed as a function of daily cumulative peak overpressure (BOP, psi) recorded by left wrist sensor. The normalization of the data was performed using the values of the DANA Rapid recorded before the training and “immediate” **(A,C)** and “post” **(B,D)** sessions. The dominant trend in the data is approximately the “33.3–66.6%” separation. The performance of the 1/3rd among all the participants decreases after the training, while the performance of the remaining 66.6% is increased. There is no obvious correlation between the DANA results and the cumulative daily pressure exposure.

In the next step we evaluated if there exists a correlation between ΔPRT or ΔGNG, and cumulative daily BOP values (Figure 8). It is clear from the Figure 8 that there is no linear correlation between the reaction time tasks and the cumulative daily peak overpressure (*R*^2^ values for all plots are below 0.1). However, there is a consistent division between the “slower” and the “faster” groups: ~1/3rd of the trainees perform the PRT and GNG tests slower after the training, while ~2/3rd of the tested population performs these tasks faster. These results are independent on the daily cumulative BOP “dose”.

## Conclusions

The environment of the 0.50 caliber marksman training was characterized in detail: we have presented the average peak overpressure (5.85 psi for the 20” barrel and 4.55 psi for the 29” barrel) and impulse (2.16 and 2.63 psi·ms, for 20” and 29” barrels, respectively) values for more than 500 measurements taken over 3 day long course. The heterogeneity of the training environment is illustrated by variability of the number of exposures per trainee expressed using different time binning methods. This variability, considering relatively constant pressure readings across the entire collected data set, is the main source of large variability in the cumulative overpressure dosage. Interestingly, barrel length seemed to have a significant and meaningful effect on BOP received by students. Upon further evaluation, we believe this is a more complex situation than is apparent. Barrel length did vary between 20- and 29-inch lengths, but muzzle devices—which direct blast and pressure from the barrel upon firing, were not accounted for. Other works also closely evaluated 0.50 caliber weapon systems and found similar effects for barrel length, but to a much smaller degree after accounting for variation from muzzle device (16). Likely, barrel length plays a role in pressure, but the extent to which may be much less than the results here indicate, and proper muzzle device selection may be a significant step in reducing these pressures.

We also evaluated the performance of the B3 blast gauges using more than 1,000 of recorded events. The regression analysis indicates that the triggering of these sensors is problematic when the peak pressure decreases, which results in considerable data loss (up to 40% at 4 psi BOP).

The neurocognitive performance was evaluated using DANA Rapid, and we established that there is a similar pattern of decrease in performance among trainees and time in training. Fatigue is likely a confounding factor among the trainees, and it might act as a co-aggravating factor contributing to the cumulative increase in the proportion of subjects with slower reaction times and processing performance over the training period. Future studies should take these observations into account and incorporate in the experimental design appropriate controls. As for speculation on why some participants “sped up” while others slowed down—a variety of potential explanations exist, of which we will discuss three. First—the fatigue factor that was previously mentioned may have played a role for some participants more than others. The age range of the course was spanned 30 years, and overall physical fitness levels varied between participants. It is possible that some were simply more tired and suffered worse performance than others. Second—as for speeding up, without a dedicated control, we can only say participants sped up from baseline, but with an appropriate control cohort we may be able to derive what an appropriate learning rate is and to understand if the rate at which these participants sped up is appropriate compared to other groups, or if their performance is actually slightly degraded. As was mentioned however, without future efforts accounting for meaningful controls, this point remains purely speculative. The final explanation could be that cumulative blast effects over the duration of the course slowed down the slower group, and still impacted the faster group, who have a suppressed learning rate we cannot detect without a control group.

This work illustrates the immense variation received in training courses and continues to point to the need for a more modular, operator focused system than static minimum safe distance (MSD) calculations. As was noted, incident pressure measurements routinely exceeded 4 psi thresholds, and varied significantly by weapon setup, shooter position, and environment. Applying a static MSD is insufficient for modern trainings. Additionally, alternative explanations do exist to evaluate changes in performance, but we cannot rule out the impact of cumulative BOP as a contributing factor that can negatively impact at least some operators' performances. The high variability in blast received across individuals in the same training course over the same duration also highlights the need for the quantification, at the individual level, of BOP exposure. The training tempo in modern courses lends itself to tailored experiences, which lead to tailored blast exposures. Simply assessing group level effects is useful, but insufficient to characterize the needs of warfighters. Finally, sensor failure makes assessments difficult. Future works need to also consider imputation methodologies that can account for missing data, or novel methodologies in general that are better at characterizing blast with incomplete information. Additional investigation is also needed to understand the increasing dispersion of results between subjects. Simply saying some individuals perform better or worse because of blast without at least a theory on root causes makes prevention and treatment near impossible. Future work should seek to determine the recovery period and longitudinal effects of heavy usage of these weapon systems.

## Data Availability

The datasets generated for this study are available on request to the corresponding author.

## Ethics Statement

All participants were provided a study briefing of research activities to be performed prior to obtaining written informed consent; the informed consent process and study activities were approved prior to data collection by the Walter Reed Army Institute of Research Intuitional Review Board.

## Author Contributions

AM, CL, ME, and GK performed the field study. MS, AM, and CL performed data analysis. MS performed data interpretation. MS, ME, CL, GK, and NC wrote the manuscript.

### Conflict of Interest Statement

The authors declare that the research was conducted in the absence of any commercial or financial relationships that could be construed as a potential conflict of interest.
